# The complete chloroplast genome sequence of *Cipadessa cinerascens*

**DOI:** 10.1080/23802359.2019.1674707

**Published:** 2019-10-11

**Authors:** Zhonghua Chen, Yi Wang

**Affiliations:** Laboratory of Forest Plant Cultivation and Utilization, Yunnan Academy of Forestry, Kunming, Yunnan, People's Republic of China

**Keywords:** *Cipadessa cinerascens*, chloroplast, Illumina sequencing, phylogenetic analysis

## Abstract

The first complete chloroplast genome sequence of *Cipadessa cinerascens* were reported in this study. The cpDNA of *C. cinerascens* is 160,590 bp in length, contains a large single copy region (LSC) of 87,855 bp and a small single copy region (SSC) of 18,623 bp, which were separated by a pair of inverted repeat (IR) regions of 27,056 bp. The genome contains 130 genes, including 85 protein-coding genes, 8 ribosomal RNA genes, and 37 transfer RNA genes. The overall GC content of the whole genome is 37.7%. Phylogenetic analysis of 11 chloroplast genomes within the family Meliaceae suggests that *C. cinerascens* is closely related to *Azadirachta indica*.

*Cipadessa cinerascens* (Pell.) Hand.-Mazz is a shrub belonging to the genus Cipadessa within the family Meliaceae and whose natural distribution in Southwest China (Peng et al. 2008). *Cipadessa cinerascens* is a famous minority-ethnic Dai folk medicine in China, the leaves and roots of *C. cinerascens* are used for the treatment of dysentery, skin itch, malaria, and burns (Lin et al. 2008). Several new triterpenoids and limonoids were isolated from *C. cinerascens* (Ren et al. 2007; Fang et al. 2009). Especially, the limonoid from *C. cinerascens* showed significant anti-HIV activities (Yu et al. 2015). *Cipadessa cinerascens* has huge medicinal value (Bandi and Lee 2012). However, there has been no genomic studies on *C. cinerascens*.

Herein, we reported and characterized the complete *C. cinerascens* plastid genome (MN126582). One *C. cinerascens* individual (specimen number: 5309270089) was collected from Lincang, Yunnan Province of China (23°23′32″ N, 98°57′22″ E). The specimen is stored at Yunnan Academy of Forestry Herbarium, Kunming, China and the accession number is YAFH0012754. DNA was extracted from its fresh leaves using DNA Plantzol Reagent (Invitrogen, Carlsbad, CA, USA).

Paired-end reads were sequenced by using Illumina HiSeq system (Illumina, San Diego, CA, USA). In total, about 25.9 million high-quality clean reads were generated with adaptors trimmed. Aligning, assembly, and annotation were conducted by CLC de novo assembler (CLC Bio, Aarhus, Denmark), BLAST, GeSeq (Tillich et al. 2017), and GENEIOUS v 11.0.5 (Biomatters Ltd, Auckland, New Zealand). To confirm the phylogenetic position of *C. cinerascens*, other 10 species of family Meliaceae from NCBI were aligned using MAFFT v.7 (Katoh and Standley 2013) and maximum likelihood (ML) bootstrap analysis was conducted using RAxML (Stamatakis 2006); bootstrap probability values were calculated from 1000 replicates. *Atalantia kwangtungensis* (MH329190) was served as the out-group.

The complete *C. cinerascens* plastid genome is a circular DNA molecule with the length of 160,590 bp, with a large single copy (LSC: 87,855 bp), small single copy (SSC: 18,623 bp), and two inverted repeats (IRa and IRb: 27,056 bp each). The overall GC content of the whole genome is 37.7% and the corresponding values of the LSC, SSC, and IR regions are 35.8, 32.2, and 42.7%, respectively. The genome contains 130 genes, including 85 protein-coding genes, 8 ribosomal RNA genes, and 37 transfer RNA genes. Phylogenetic analysis showed that *C. cinerascens* clustered together with *Azadirachta indica*, which indicated the phylogenesis classification of *C. cinerascens* (Figure 1). The determination of the complete plastid genome sequences provided new molecular data to illuminate the Meliaceae evolution.

**Figure 1.**
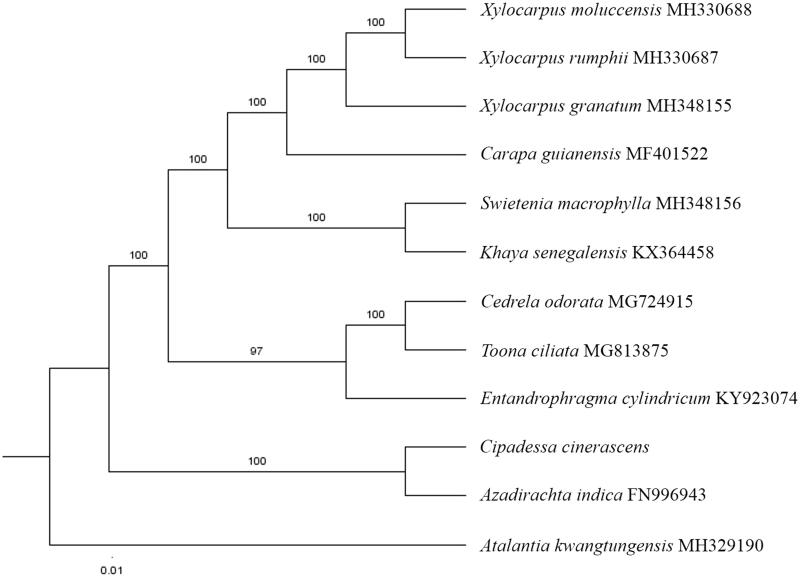
The maximum-likelihood tree based on the 11 chloroplast genomes of family Meliaceae. The bootstrap value based on 1000 replicates is shown on each node.
